# TFAP2C-Mediated lncRNA PCAT1 Inhibits Ferroptosis in Docetaxel-Resistant Prostate Cancer Through c-Myc/miR-25-3p/SLC7A11 Signaling

**DOI:** 10.3389/fonc.2022.862015

**Published:** 2022-03-23

**Authors:** Xingkang Jiang, Shanqi Guo, Mengyao Xu, Baojie Ma, Ranlu Liu, Yong Xu, Yangyi Zhang

**Affiliations:** ^1^ Department of Urology, Tianjin Institute of Urology, The Second Hospital of Tianjin Medical University, Tianjin, China; ^2^ The International Collaborative Laboratory for Biological Medicine of the Ministry of Education, The School of Medicine, Nankai University, Tianjin, China; ^3^ Department of Oncology, First Teaching Hospital of Tianjin University of Traditional Chinese Medicine, National Clinical Research Center for Chinese Medicine Acupuncture and Moxibustion, Tianjin, China; ^4^ Department of Oncology, Beijing Shijitan Hospital of Capital Medical University, Beijing, China

**Keywords:** LncRNA, docetaxel, ferroptosis, prostate cancer, PCAT1

## Abstract

Recent evidence has shown that the induction of ferroptosis is a new therapeutic strategy for advanced prostate cancer (PCa) when used as a monotherapy or in combination with second-generation antiandrogens. However, whether ferroptosis inducers are effective against docetaxel-resistant PCa remains unclear. In addition, the biological role and intrinsic regulatory mechanisms of long noncoding RNAs (lncRNAs) in ferroptosis and chemoresistance are not well understood. In this study, we established two acquired docetaxel-resistant PCa cell lines and found that docetaxel-resistant PCa cells developed tolerance toward ferroptosis. In addition, dysregulated lncRNAs in drug-resistant and -sensitive PCa cells were identified by RNA sequencing, and we identified that prostate cancer-associated transcript 1 (*PCAT1*) was highly expressed in the docetaxel-resistant PCa cell lines and clinical samples. Overexpression of *PCAT1* inhibited ferroptosis and increased docetaxel resistance, which could be attenuated by *PCAT1* knockdown. Furthermore, we revealed that *PCAT1* inhibited ferroptosis by activating solute carrier family 7-member 11 (SLC7A11) expression *via* reducing iron accumulation and subsequent oxidative damage. Mechanistically, we demonstrated that *PCAT1* interacted with c-Myc and increased its protein stability using nucleotides 1093-1367 of *PCAT1* and 151-202 amino acids of c-Myc protein, thereby transcriptionally promoting SLC7A11 expression. In addition, *PCAT1* facilitated SLC7A11 expression by competing for microRNA-25-3p. Finally, transcription factor AP-2 gamma (TFAP2C) activated *PCAT1* expression at the transcriptional level to reduce ferroptosis susceptibility and enhance chemoresistance. Collectively, our findings demonstrated that TFAP2C-induced *PCAT1* promotes chemoresistance by blocking ferroptotic cell death through c-Myc/miR-25-3p/SLC7A11 signaling.

## Introduction

Prostate cancer (PCa) was the second most common cancer and the fifth leading cause of cancer death among men worldwide in 2021 (1). Advances in the treatments for PCa patients have improved over the last decades, including radical prostatectomy, radiotherapy, and androgen deprivation therapy. However, many patients experience relapse after initial therapy. Although docetaxel (DTX)-based chemotherapy is currently recommended as one of the first-line treatments for metastatic PCa patients, most patients eventually progress because of inherent or acquired drug resistance (2). A variety of mechanisms have been proposed to contribute to DTX resistance, such as multidrug resistance mechanisms, alterations in β-tubulin isotypes, mutations in tumor suppressor proteins, inhibition of the apoptotic cell death pathway and misregulated androgen signaling (3, 4). Therefore, an understanding of how these multiple mechanisms contribute to chemoresistance will be important when developing new treatment strategies for advanced PCa.

Ferroptosis is a novel form of regulated cell death *via* iron-dependent accumulation and lipid peroxidation, which is morphologically, biochemically and genetically distinct from apoptosis, necrosis, and autophagy (5). The key molecules related to ferroptosis include solute carrier family 7 member 11 (SLC7A11) and glutathione peroxidase (GPX4), an essential regulator of ferroptosis that suppresses lipid peroxidation (6). Various diseases, including cancer, have been linked to abnormal ferroptosis, and inhibition of SLC7A11 and GPX4 may eradicate cancer cell resistant to chemotherapy, targeted therapy or radiotherapy. For example, genetic ablation of SLC7A11 in pancreatic ductal adenocarcinoma cells exhibits amino acid stress and glutathione (GSH) depletion and potentiates the cytotoxic effects of both gemcitabine and cisplatin (7). In addition, GPX4 ablation abolished ionizing radiation-induced ferroptosis and promoted radioresistance (8). Recently, Ghoochani et al. reported that ferroptosis inducers independently and in combination with second-generation antiandrogens are novel therapeutic strategies for advanced cancer (9). In addition, Bordini and colleagues also demonstrated that iron exacerbates oxidative damage and cell death and thus strengthens the efficacy of antiandrogen therapy in PCa cells and xenografts (10). However, the potential interplay between DTX resistance and ferroptosis has not been fully investigated. In other words, whether ferroptosis inducers are still effective against DTX-resistant PCa needs to be further explored.

Long noncoding RNAs (lncRNAs) are a group of transcripts that are longer than 200 nucleotides. Previous studies indicated that aberrant expression of lncRNAs was associated with cell growth, cell apoptosis, and drug resistance (11). For example, Zhou et al. found that PVT1 promotes gemcitabine resistance in pancreatic cancer by activating the Wnt/β-catenin and autophagy pathways by modulating the miR-619-5p/Pygo2 and miR-619-5p/ATG14 axes (12). In addition, LINC00680 promotes hepatocellular carcinoma stemness properties and decreases chemosensitivity by sponging miR-568 to activate AKT3 expression (13). Our previous studies and others also revealed that lncRNAs play critical roles in the chemoresistance of PCa, including HOTAIR, NEAT1, DANCR, MALAT1 and CASC2 (14). However, the expression profiling and biological functions of lncRNAs in DTX-resistant PCa remain incompletely understood. Moreover, recent studies have also demonstrated that lncRNAs are associated with ferroptosis among various tumors (15). For example, lncRNA P53RRA can interact with G3BP1 in the cytoplasm, thus promoting ferroptosis and apoptosis in cancer *via* nuclear sequestration of p53 (16). In 2019, Wang et al. identified that LINC00336 acts as a crucial inhibitor of ferroptosis in non-small cell lung cancer by decreasing intracellular iron levels and lipid reactive oxygen species (ROS) content (17). Our recent study also illustrated that lncRNA OIP5-AS1 inhibits ferroptosis in PCa with long-term cadmium exposure through miR-128-3p/SLC7A11 signaling (18). Nevertheless, the potential regulatory role of lncRNAs in ferroptosis in PCa cells and whether they affect the efficacy of chemoresistance remain unclear.

To unravel these questions, we established two acquired DTX-resistant PCa cell lines and found that DTX-resistant PCa cells develop tolerance toward ferroptosis. In addition, we further screened the differentially expressed lncRNAs in DTX-resistant and -sensitive PCa cells by RNA sequencing and identified that prostate cancer-associated transcript 1 (PCAT1) was robustly upregulated in DTX-resistant PCa cells and clinical samples. Further experiments demonstrated that PCAT1 inhibited ferroptosis by activating SLC7A11 expression. Mechanistic studies revealed that PCAT1 promoted the expression of SLC7A11 by binding with the c-Myc protein and sponging miR-25-3p. In addition, transcription factor AP-2 gamma (TFAP2C) activated PCAT1 expression at the transcriptional level to reduce ferroptosis susceptibility and enhance chemoresistance.

## Materials and Methods

### Clinical Samples

Human PCa samples and adjacent tissues used in this study were obtained from 42 localized PCa patients who underwent radical prostatectomy at the Second Hospital of Tianjin Medical University, as our previously described (14). In addition, we also obtained the serum of 33 metastatic PCa patients who underwent at least 4 cycles of DTX-based chemotherapy from January 2020 to January 2021. This study was approved by the Ethics Committee of the Second Hospital of Tianjin Medical University, and written informed consent was obtained from each patient. Clinical tissues and serum samples were snap-frozen immediately in liquid nitrogen and stored until use. DTX resistance is defined as progression (i.e. PSA level or imaging examination) after at least 4 cycles of standard DTX-based chemotherapy.

### Cell Lines and Reagents

The human prostate carcinoma cell lines PC3 and 22RV1 were purchased from the National Collection of Authenticated Cell Culture (Shanghai, China) and maintained in DMEM/F12 medium supplemented with 10% fetal bovine serum (Biological Industries, Israel) and 1% antibiotics (penicillin and streptomycin, HyClone, USA). As previously described, DTX-resistant cell lines (PC3/DR and 22RV1/DR) were developed by stepwise increased DTX concentrations over a period of 8-9 months (19, 20). DTX, Erastin, Ferrostatin-1 (Fer-1),CHX and Borz were purchased from MedChemExpress (China).

### RNA-Sequencing Analysis

Total RNA used for RNA sequencing was isolated from three independent groups of PC3 and PC3/DR cells using TRIzol reagents (Invitrogen, USA). High-quality RNA samples were subjected to library construction, and then the library was sequenced on an Illumina NovaSeq™ 6000 following the standard procedures of Geneseed (China). In addition, the mRNA library of *PCAT1*-knockdown PC3/DR cells and control PC3/DR cells was sequenced on an Illumina NovaSeq™ 6000 following the vendor’s recommended protocol (LC-Bio, China). The differentially expressed lncRNAs and mRNAs were selected with fold change > 2 or fold change < 0.5 and *p* value < 0.05 by the R package edgeR.

### Cell Viability and Colony Formation

Cell viability was analyzed by a CCK-8 assay (Dojindo, Japan) according to the manufacturer’s protocol. For cell colony formation, 2000 cells were seeded into the wells of 6-well plates and cultured in specific culture media. After 2 weeks, the cells were fixed with ethanol and stained with 0.1% crystal violet (Solarbio, China). The number of visible colonies was counted using ImageJ software.

### Lipid ROS Production, Lipid Peroxidase Content and GSH Levels

Lipid ROS production was detected using an ROS Assay Kit (Beyotime, China). Lipid peroxidase was assessed by a Liperfluo Assay Kit (Dojindo, Japan), and the GSH level was evaluated by a GSH assay kit (Nanjing Jiancheng, China) following the manufacturer’s protocol.

### Intracellular Iron Content, Mitochondrial Membrane Potential and Transmission Electron Microscope

Intracellular total iron and ferrous iron concentrations were detected by an iron assay kit following the manufacturer’s protocol (Sigma-Aldrich, USA). A FerroOrange assay (Dojindo, Japan) was used to detect the ferrous iron level, and the mitochondrial membrane potential was detected by a Mitochondrial Membrane Potential Assay Kit with JC-1 (Beyotime, China). The TEM samples were prepared using our previously described method, and ultra-structural images were captured with the transmission electron micorscope (JEM-1011, Japan) (18).

### Quantitative Real-Time PCR

Total RNA was isolated from cells and clinical samples using TRIzol reagent (Invitrogen, USA). Reverse transcription was performed to synthesize cDNA using the lncRcute and miRcute Kit (Tiangen, China). qRT-PCR was conducted using Hieff SYBR Green Master Mix (Yeasen, China). The expression of lncRNAs, mRNAs and miRNAs was calculated and normalized to the internal control β-actin by using the standard 2^-ΔΔCt^ method. The qRT-PCR primer sequences are listed in **Table S1**.

### Western Blot Analysis

Cells were lysed on ice with RIPA buffer (Beyotime, China) containing 1 × protease inhibitor cocktail (Sigma-Aldrich, USA). Equal amounts of protein samples (20 μg) were separated by 10% SDS-PAGE gels and then transferred to PVDF membranes. The membranes were incubated with primary antibodies and the associated secondary rabbit or mouse antibodies. The primary antibodies were as follows: SLC7A11 (1:1000, Abcam, USA, Cat. ab175186), GPX4 (1:1000, Abcam, USA, Cat. ab125066), TFAP2C (1:1000, Proteintech, China, Cat. 14572-1-AP), c-Myc (1:1000, Proteintech, Cat. 67447-1-Ig) and β-actin (1:1000, Proteintech, China, Cat. 66009-1-Ig).

### Cell Transfection and Infection

Full-length *PCAT1*, SLC7A11, c-Myc, TFAP2C and their negative control cDNA were cloned into the pcDNA3.1 vector (Invitrogen, USA). siRNAs molecules specifically targeting *PCAT1*, SLC7A11 c-Myc and TFAP2C were designed and synthesized by RiboBio (Guangzhou, China). miRNA mimics and inhibitors of miR-25-3p were also purchased from RiboBio (Guangzhou, China). Lipofectamine 2000 Reagent (Invitrogen, USA) was used to transiently transfect these reagents. In addition, a GV112 lentivirus vector (GeneChem, China) was used to generate short hairpin RNAs (shRNAs) against *PCAT1* (labeled as shPCAT1 #1) and a negative control (labeled as shNC). The lentiviral packaging for the *PCAT1* expression plasmids and empty control were generated with a lentiviral packaging kit (GeneChem, China). The infected PCa cells were selected with 2 μg/mL puromycin (Sigma-Aldrich, USA) for up to 2 weeks. The siRNA sequences are presented in **Table S1**.

### RNA Pulldown and Silver Staining

The RNA pull-down assay was performed by a Pierce™ Magnetic RNA-Protein Pull-Down Kit (Thermo Scientific, USA). Biotin-labeled full-length and short-length *PCAT1* probes were synthesized by GenePharma (Shanghai, China). In brief, the labeled RNA probe was incubated with streptavidin magnetic beads and washed with 20 mM Tris. Then, the master mix of the RNA-protein binding reaction was incubated with RNA-bound beads. The lncRNA-interacting proteins were eluted with 1× SDS loading buffer and further separated by 10% SDS-PAGE gels. The Fast Silver Stain Kit (Beyotime, China) was used to visualize the associated proteins.

### RNA Immunoprecipitation

The RIP assay was performed using a Magna RIP RNA-Binding Protein Immunoprecipitation Kit (Millipore, USA) according to the manufacturer’s instructions. In brief, PC3/DR cells were harvested and lysed in RIP lysis buffer and then incubated with magnetic beads coated with anti-c-Myc and anti-IgG antibodies. Then, the coprecipitated RNAs were extracted and evaluated by qRT-PCR.

### Dual-Luciferase Reporter Assay

The wild-type and mutant *PCAT1* and SLC7A11 3’UTRs were cloned into a pmiR-RB-Report™ vector (RiboBio, China). Then, PC3 cells were plated on a 96-well plate and cotransfected with wild-type and mutant luciferase reporter plasmids and miR-25-3p mimic. In addition, the promoter segments of *PCAT1* and SLC7A11 were cloned into the pGL3-basic vector (Promega, USA). The pGL3-*PCAT1* and pGL3-SLC7A11 vectors were cotransfected with TFAP2C or c-Myc plasmids in PC3 cells. The relative luciferase activity was measured by the Dual Luciferase Assay System according to the manufacturer’s protocol (Promega, USA).

### Chromatin Immunoprecipitation

ChIP assays were performed using a ChIP kit following the manufacturer’s instructions. Briefly, cells were crosslinked with formaldehyde and sonicated to 200-1000 bp. Immunoprecipitation was conducted with an anti-c-Myc, antiTFAP2C or anti-IgG control. Precipitated DNA was amplified by qPCR. The ChIP-qPCR sequences are listed in **Table S1**.

### Animal Experiments

All mice were housed in a specific pathogen-free environment at the Second Hospital of Tianjin Medical University, and experimental procedures were approved by the Tianjin Medical University Institutional Animal Care and Use Committee. PC3 and PC3/DR cells (5×10^6^ cells) were subcutaneously injected into each flank of six-week-old male BALB/c nude mice (HFK Biotech, China). When the tumor volume reached 100 mm^3^, the mice were treated with Dimethyl Sulfoxide (DMSO) alone, DTX (5 mg/kg body weight, every two days) with DMSO or erastin (20 mg/kg body weight in 20 μl DMSO plus 130 μl corn oil, daily) by intraperitoneal injection, as previously described (9, 20). The tumor volumes of the mice were assessed by measuring the length and width every three days. After 4 weeks, all mice were sacrificed, and the tumors and metastatic tissues were harvested for further analysis.

### Immunohistochemistry Staining

IHC was conducted on tumor samples from xenograft mice following standard methods. The tumor specimens were fixed in formalin, embedded in paraffin and then cut into 4 μm slices. After dewaxing, rehydration and antigen retrieval, the slides were incubated with specific primary antibodies against Ki-67 (1:5000, Proteintech, China) and SLC7A11 (1:1000, Proteintech, China). After incubation with the corresponding secondary antibodies, the sections were stained with diaminobenzidine and counterstained with hematoxylin. Representative images were photographed using an Olympus light microscope.

### Statistical Analysis

All statistical analyses were performed using GraphPad Prism 8.0 (GraphPad Software, USA) and SPSS software 17.0 (IBM, USA). The data are presented as the mean ± standard deviation (SD) from three independent experiments. Statistical significance was analyzed using Student’s *t*-test or two-way ANOVA tests. The correlation between *PCAT1* and TFAP2C expression levels was determined by using Pearson’s correlation coefficient. All data were considered statistically significant at a *p* value < 0.05.

## Results

### DTX-Resistant PCa Cells Develop Tolerance Toward Ferroptosis

**Figure 1:**
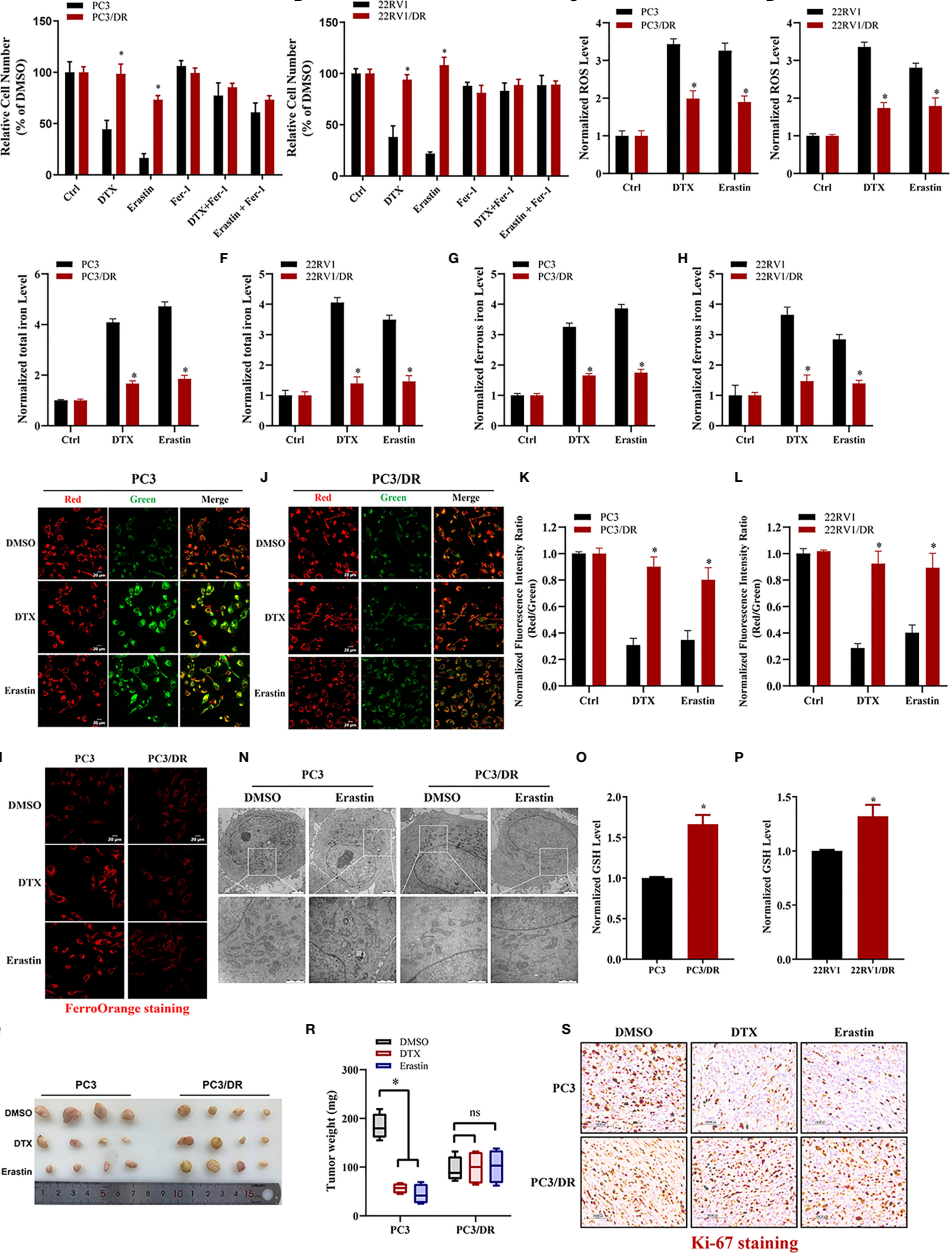
DTX-resistant PCa cells develop tolerance toward ferroptosis. **(A, B)** CCK-8 assay was used to determine the response of PC3 and PC3/DR cells, 22RV1 and 22RV1/DR cells to DTX (10nM), erastin (5μM) and/or ferrostatin-1 (5μM). **(C, D)** Lipid ROS content was analyzed in PC3 and PC3/DR, 22RV1 and 22RV1/DR cells under DTX (10nM) or erastin (5μM) treatment. **(E–H)** Levels of total iron, ferrous iron concentration were evaluated in PC3 and PC3/DR cells, 22RV1 and 22RV1/DR cells upon DTX (10nM) or erastin (5μM) exposure. **(I–L)** The mitochondrial membrane potential were assessed by JC-1 assays in PC3 and PC3/DR cells, 22RV1 and 22RV1/DR cells after DTX (10nM) or erastin (5μM) treatment. **(M)** FerroOrange staining showed that ferrous iron content (red) in PC3 and PC3/DR cells under DTX (10nM) or erastin (5μM) treatment. **(N)** The morphological changes of mitochondria in PC3 and PC3/DR cells by erastin (5μM) were observed by transmission electron microscope. **(O, P)** The GSH content was detected in PC3 and and PC3/DR cells, 22RV1 and 22RV1/DR cells. **(Q, R)** Representative images of xenograft tumors of PC3 and PC3/DR cells after treated with either DTX (5 mg/kg body weight), erastin (20 mg/kg body weight) or DMSO, and the tumor weight were further measured accordingly. The statistical significance of tumor weight was calculated by using two-way ANOVA. **(S)** H&E and IHC staining were performed to evaluate the tissue morphology and Ki-67 expression in each group. The data are presented as the mean ± S.D. of at least three independent experiments. *P < 0.05. ns, no significance.

To clarify whether ferroptosis inducer can also inhibit DTX-resistant PCa, we generated two acquired DTX-resistant cell lines (PC3/DR and 22RV1/DR) derived from castration-resistant PCa cells (PC3 and 22RV1) as our previously described (19, 20). DTX resistance was further validated by cell viability and colony formation assays (**Figure S1A–D**). Subsequently, we assessed the susceptibility to ferroptosis in DTX-resistant PCa cells and their drug-naïve parental cells following treatment with the ferroptosis inducer erastin. As expected, the growth of PC3 and 22RV1 cells was significantly inhibited by DTX and erastin, and cell growth inhibition was reversed by the ferroptosis inhibitor Fer-1 (**Figures 1A, B** **)**. Exposure to DTX or erastin in PC3 and 22RV1 cells greatly increased the lipid ROS levels and intracellular total iron and ferrous iron content (**Figures 1C–H**), whereas the mitochondrial membrane potential was decreased upon DTX and erastin treatment (**Figures 1I–L**). Intriguingly, we found that the cytotoxicity of DTX and erastin was restricted in PC3/DR and 22RV1/DR cells (**Figures 1A, B** **)**. In addition, PC3/DR and 22RV1/DR cells had a ferroptosis resistance phenotype against erastin and DTX compared with PC3 and 22RV1 cells (**Figures 1C–L**). FerrOrange staining assays also revealed that PC3 and 22RV1 cells had higher ferrous iron levels after DTX and erastin treatment than PC3/DR and 22RV1/DR cells (**Figure 1M**). When PC3 and PC3/DR cells were treated with erastin, the morphological features of mitochondria involved a larger size, decreased membrane density and cristae thinning in PC3 cells, while the morphology of mitochondria was not changed in PC3/DR cells (**Figure 1N**). Moreover, the GSH content was higher in DTX-resistant PCa cells than in DTX-sensitive cells (**Figures 1O, P** **)**. To further assess the therapeutic potential of erastin in DTX-resistant PCa cells, we treated BALB/c nude mice bearing PC3 and PC3/DR cells with erastin, DTX and DMSO when the tumor volume reached 100 mm^3^. As shown in **Figures 1Q, R**, exposure to erastin or DTX significantly delayed the tumor growth of PC3 xenografts by tumor weight compared to treatment with DMSO. Consistent with the *in vitro* findings, we did not observe any significant differences in tumor growth in PC3/DR xenografts treated with erastin or DTX (**Figures 1Q, R** **)**. In addition, Immunohistochemistry (IHC) staining revealed that the tissue morphology and Ki-67 levels in the PC3/DR group were similar upon DTX and erastin treatment (**Figure 1S**). These results indicated that DTX-resistant PCa cells have the ability to inhibit ferroptosis *in vitro* and *in vivo*.

### PCAT1 Overexpression Increases DTX Resistance and Inhibits Ferroptosis

**Figure 2:**
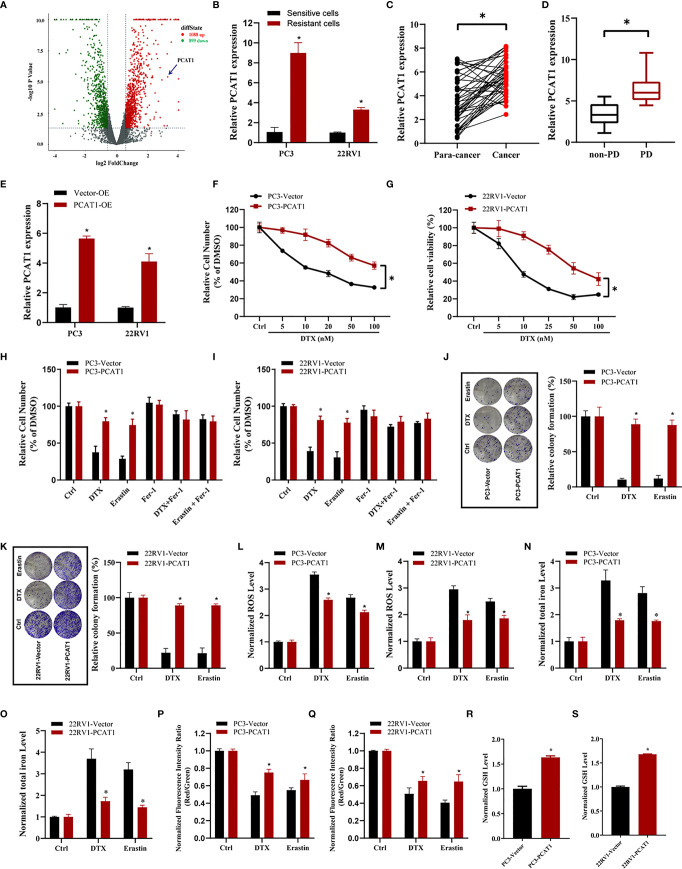
PCAT1 overexpression increases DTX resistance and inhibits ferroptosis. **(A)** Volcano plot representative the differentially expressed lncRNAs in DTX-resistant PC3/DR cells and counterpart PC3 cells. The red and green represent the upregulated and downregulated lncRNAs. **(B)** The expression of PCAT1 was assessed by qRT-PCR in DTX-resistant PCa cells (PC3/DR and 22RV1/DR) and their sensitive cells (PC3 and 22RV1). **(C)** The relative expression of PCAT1 was evaluated by qRT-PCR in 42 pairs of PCa tissues and para-cancer samples. **(D)** qRT-PCR analysis of PCAT1 in the pre-therapy plasma of PCa patients with progressive disease (PD) (n=15) or non-PD (n=18) after DTX chemotherapy. **(E)** The efficiency of PCAT1 overexpression in PC3 and 22RV1 cells was detected by qRT-PCR. **(F, G)** The cell viability was evaluated in PC3 and 22RV1 cells after transfected with PCAT1 upon different dosage of DTX treatment for 72 hours. **(H, I)** CCK-8 assay was used to analyze the response of PCAT1-induced PC3 and 22RV1 cells to DTX, erastin and ferrostatin-1. **(J, K)** Colony formation assay was used to assess cell survival in PCAT1-overexpressed PC3 and 22RV1 cells after DTX or erastin treatment. **(L, M)** Lipid ROS content, **(N, O)** total iron concentration, **(P, Q)** the mitochondrial membrane potential were evaluated in PC3 and 22RV1 cells after PCAT1 overexpression upon DTX and erastin treatment. **(R, S)** The GSH content was assessed in PCAT1-induced PC3 and 22RV1 cells. The data are presented as the means ± S.D. of at least three independent experiments. *P < 0.05.

To elucidate the potential role of lncRNAs mediating PCa chemoresistance, we performed a comprehensive analysis of lncRNAs using whole transcriptome sequencing to compare DTX-resistant PC3/DR cells with sensitive parental PC3 cells. Subsequent differential expression analysis revealed 1 088 upregulated lncRNAs and 899 downregulated lncRNAs in PC3/DR cells compared to PC3 cells (**Figure 2A**). Subsequently, we selected the top 20 upregulated lncRNAs according to the inclusion criteria for further validation by quantitative reverse transcription-polymerase chain reaction (qRT-PCR) (**Figure S2A**). Among these lncRNAs, we observed that *PCAT1* expression was greatly upregulated in PC3/DR and 22RV1/DR cells compared to PC3 and 22RV1 cells by qRT-PCR (**Figure 2B**). To validate the clinical significance of *PCAT1* expression in PCa, we evaluated the expression level of *PCAT1* in PCa samples. Consistent with our previous study, the expression of *PCAT1* was found to be significantly upregulated in tumors compared with adjacent normal tissues (**Figure 2C**) (21). Moreover, we found that the expression level of *PCAT1* in pretherapy plasma was higher in metastatic PCa patients who suffered from progressive disease (PD) after at least 4 cycles of DTX chemotherapy than in those without PD (**Figure 2D**). Together, the above data indicated that elevated *PCAT1* expression might be associated with DTX resistance in PCa.

To further explore whether *PCAT1* mediates DTX-induced ferroptosis in PCa cells, we ectopically expressed *PCAT1* in PC3 and 22RV1 cells (**Figure 2E**). Cell viability assays showed that OE of *PCAT1* enhanced DTX resistance in PC3 and 22RV1 cells (**Figures 2F, G** **)**. In addition, we also found that *PCAT1* OE reversed the erastin-induced and DTX-induced growth inhibition of PC3 and 22RV1 cells by CCK-8 assay and colony formation assay (**Figures 2H–K**). Additionally, we further assessed the phenotype of ferroptosis in *PCAT1*-transfected PC3 and 22RV1 cells. As shown in **Figures 2L–Q**, lipid ROS production and intracellular iron content levels were greatly decreased upon DTX and erastin treatment, whereas mitochondrial membrane potential was increased. In addition, we also found that GSH levels were increased in PC3 and 22RV1 cells after *PCAT1* transfection (**Figures 2R, S**). Taken together, our results demonstrated that upregulation of *PCAT1* could increase DTX resistance and inhibit ferroptosis.

### PCAT1 Knockdown Promotes Ferroptosis and Impairs DTX Resistance

**Figure 3:**
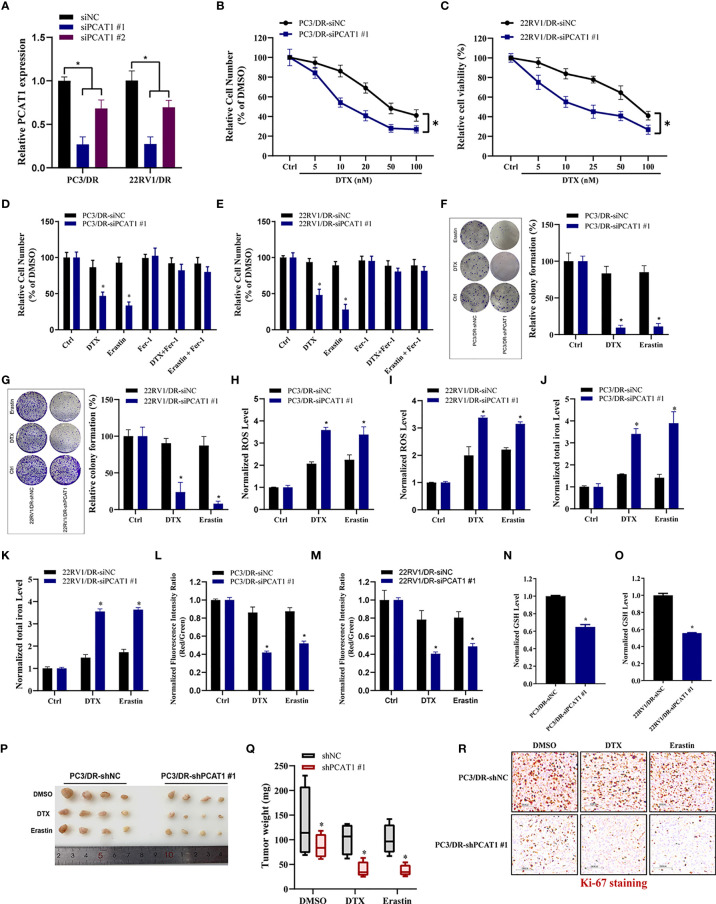
PCAT1 knockdown promotes ferroptosis and impairs DTX tolerance. **(A)** The relative expression of PCAT1 was assessed by qRT-PCR in PC3/DR and 22RV1/DR cells after transfected with siPCAT1 and negative control siRNA. **(B, C)** The cell viability was evaluated in PC3/DR and 22RV1/DR cells after PCAT1 knockdown upon different dosage of DTX treatment for 72 hours. **(D, E)** CCK-8 assay was used to analyze the response of PCAT1-knockdown PC3/DR and 22RV1/DR cells to DTX, erastin and ferrostatin-1. **(F, G)** Colony formation assay was used to evaluate the cell growth in PC3/DR and 22RV1/DR cells after PCAT1 knockdown under DTX and erastin treatment. **(H, I)** Lipid ROS content, **(J, K)** total iron concentration, **(L, M)** the mitochondrial membrane potential were evaluated in PC3/DR and 22RV1/DR cells after PCAT1 knockdown upon DTX and erastin exposure. **(N, O)** The GSH content was assessed in PCAT1-knockdown PC3/DR and 22RV1/DR cells. **(P)** Representative images of xenograft tumors of PCAT1-knockdown PC3/DR cells after treated with DTX, erastin and DMSO, and **(Q)** The tumor weight were measured accordingly. **(R)** H&E and IHC staining were performed to evaluate tissue morphology and Ki-67 expression in PCAT1 knockdown tumors and their control tissues. The data are presented as the means ± S.D. of at least three independent experiments. *P < 0.05.

To further evaluate the ferroptotic process of *PCAT1* in DTX-resistant PCa cells, we performed transient KD of *PCAT1* in PC3/DR and 22RV1/DR cells by using small interfering RNA (siRNA) molecules. The efficiency of KD was verified by qRT-PCR assay (**Figure 3A**). Inhibition of *PCAT1* in PC3/DR and 22RV1/DR cells significantly suppressed DTX resistance (**Figures 3B, C** **)**. Moreover, *PCAT1* KD increased the erastin-induced and DTX-induced cytotoxicity of PC3/DR and 22RV1/DR cells (**Figures 3D, E**). At the same time, the cell growth inhibition of DTX and erastin could be reversed by Fer-1 in *PCAT1* KD cells (**Figures 3D, E** **)**. Colony formation assays also revealed that *PCAT1* KD increased the cytotoxicity of erastin or DTX in PC3/DR and 22RV1/DR cells (**Figures 3F, G** **)**. In addition, we found that inhibition of *PCAT1* increased lipid ROS production and intracellular iron content, whereas mitochondrial membrane potential was decreased (**Figures 3H–M**). GSH levels were increased in PC3/DR and 22RV1/DR cells after *PCAT1* KD (**Figures 3N, O** **)**. In addition, we also generated xenografts by subcutaneous injection of PC3/DR cells with stable KD of *PCAT1* and control cells followed by intraperitoneal treatment with DTX and erastin. Thus, we constructed PCAT1 shRNA (i.e. shPCAT1 #1) using the sequence of siRNA #1 to stable KD the expression of PCAT1 in PC3/DR cells (**Figure S3** **)**. Compared with the control group, xenograft tumor weights were significantly lower in the *PCAT1* KD group (**Figures 3P, Q** **)**. Of note, the *PCAT1* KD group treated with erastin or DTX treatment showed the slowest tumor growth among all groups (**Figures 3P, Q** **)**. In addition, IHC staining also revealed that the malignancy of tumor cells was significantly suppressed in the *PCAT1* KD group (**Figure 3R**). Taken together, these results suggested that inhibition of *PCAT1* impairs DTX resistance and promotes ferroptosis *in vitro* and *in vivo*.

### PCAT1 Mediates Ferroptosis Resistance by Activating SLC7A11 Expression

**Figure 4:**
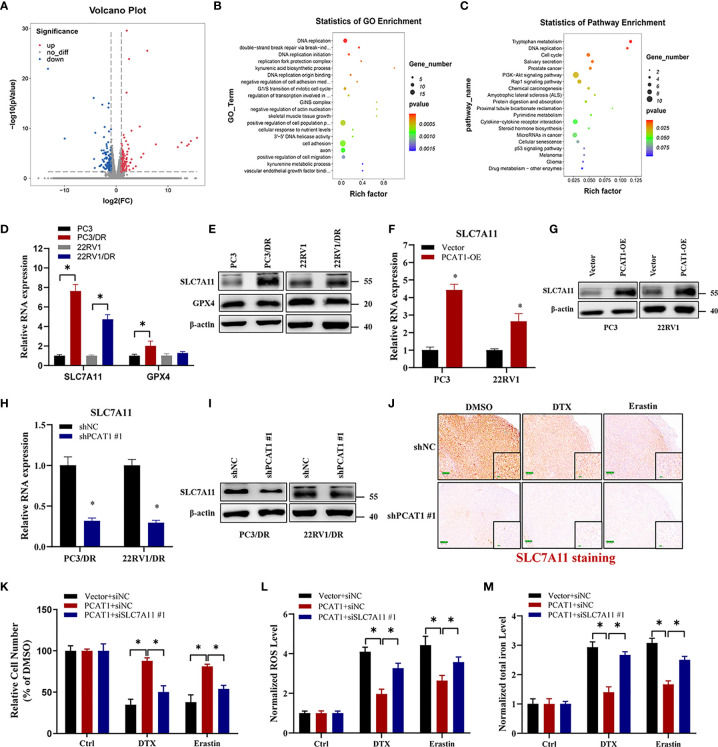
PCAT1 mediates ferroptosis resistance by activating SLC7A11 expression. **(A)** The Volcano map representative the differentially expressed mRNAs in PC3/DR cells after PCAT1 knockdown. The red and green represent the upregulated and downregulated mRNAs. **(B, C)** GO and KEGG pathway analysis showed significant functional enrichment of DNA replication, double-strand break repair, oxidation-reduction process, drug metabolism and microRNA in cancer. **(D, E)** The mRNA and protein levels of SLC7A11 and GPX4 were evaluated by qRT-PCR and Western blot in DTX-resistant PCa cells (PC3/DR and 22RV1/DR) compared with their sensitive cells (PC3 and 22RV1). **(F, G)** The relative expression of SLC7A11 was evaluated by qRT-PCR and Western blot in PC3 and 22RV1 cells after PCAT1 overexpression, and **(H, I)** PC3/DR and 22RV1/DR cells after PCAT1 knockdown. **(J)** The expression of SLC7A11 was detected by IHC in xenografts mice model unpon PCAT1 knockdown. **(K–M)** The cell viability, lipid ROS content and total iron concentration were detected in PCAT1-overexpressed PC3 cells after co-transfected with si-SLC7A11 and control siRNA under DTX and erastin treatment. The data are presented as the means ± S.D. of at least three independent experiments. *P < 0.05.

Given the putative role of *PCAT1* in DTX-resistant PCa, we evaluated the mRNA expression profiles in PC3/DR cells after KD of *PCAT1* with siRNA. As shown in **Figure 4A**, RNA-sequencing results revealed a total of 332 genes that were differentially expressed (168 upregulated genes and 164 downregulated genes) (fold change >2, *p <*0.05). Gene Ontology and Kyoto Encyclopedia of Genes and Genomes (KEGG) analysis showed significant functional enrichment of DNA replication, double-strand break repair, oxidation-reduction process, drug metabolism and microRNA (miRNA) in cancer (**Figures 4B, C** **)**. To identify the key regulators of ferroptosis in DTX-resistant PCa, we detected the expression of SLC7A11 and GPX4 by qRT-PCR and Western blot assay. As shown in **Figures 4D, E**, the mRNA and protein levels of SLC7A11 were greatly upregulated in PC3/DR and 22RV1/DR cells, whereas no significant difference was found in GPX4 expression. To further elucidate whether *PCAT1* regulates SLC7A11 expression, we detected the expression of SLC7A11 after *PCAT1* KD or OE. As expected, the mRNA and protein levels of SLC7A11 were greatly elevated in *PCAT1*-transfected PC3 and 22RV1 cells (**Figures 4F, G** **)**. In addition, inhibition of *PCAT1* decreased the mRNA and protein levels of SLC7A11 in PC3/DR and 22RV1/DR cells (**Figures 4H–I**). IHC staining also showed that *PCAT1* knockdown decreased the SLC7A11 expression in xenografts mice model (**Figure 4J**). Moreover, we also performed rescue experiments to explain why *PCAT1* inhibits ferroptosis by regulating SLC7A11 expression. As shown in **Figure 4K**, inhibition of SLC7A11 increased the cytotoxicity of DTX and erastin in *PCAT1*-transfected PC3 cells. In addition, intracellular ROS levels and total iron content were also increased (**Figures 4L, M** **)**. Collectively, the above data together indicated that *PCAT1* inhibits ferroptosis by activating SLC7A11 expression in DTX-resistant PCa cells.

### PCAT1 Physically Interacts With C-Myc and Increases Its Protein Stability

**Figure 5:**
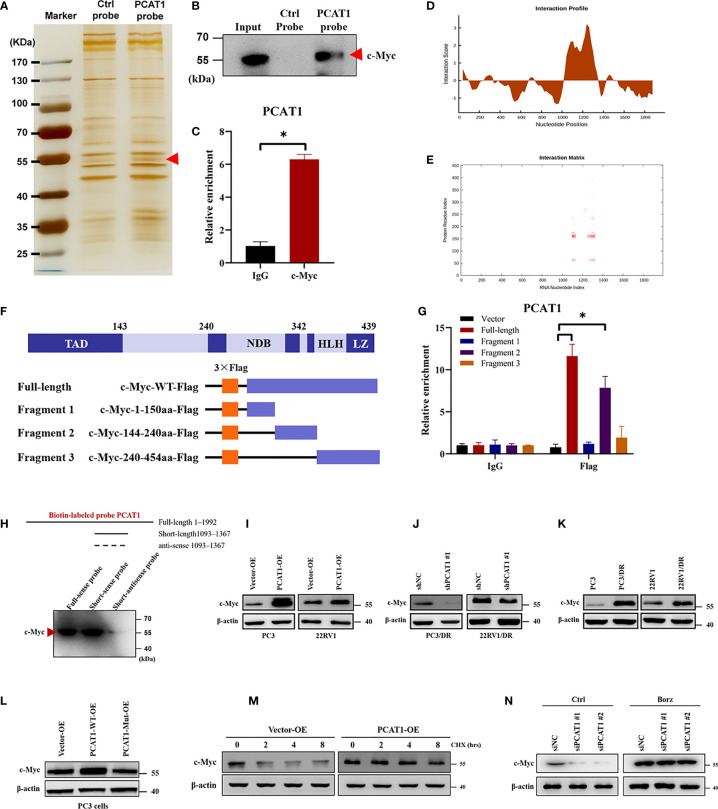
PCAT1 physically interacts with c-Myc and increases its protein stability. **(A, B)** RNA-protein pulldown experiment was performed using the specific biotin-labeled PCAT1 probe in PC3/DR lysates, followed by silver staining, and the protein bands were analyzed by Western blot. **(C)** RIP assay was executed in PC3/DR lysates using anti-cMyc or anti-IgG, then the enrichment of PCAT1 was detected by qRT-PCR. **(D, E)** CatRAPID fragments module prediction of the interaction profile and matrix between PCAT1 and c-Myc protein. **(F)** The different fragments plasmids of the c-Myc protein were illustrated blew. TAD, transactivation domain. NDB, non-specific DNA binding domain. HLH, helix loop helix domain. LZ, leucine zipper domain. **(G)** The interaction of c-Myc protein regions with PCAT1 in PC3 cells was determined by using a RIP assay. **(H)** RNA pulldown and Western blot were used to verify the possible binding of c-Myc protein by using a short biotin-labeled probe containing 1093–1367 nt of PCAT1. (**(I, J)** Western blot was used to evaluate the protein level of c-Myc upon PCAT1 overexpression or knockdown. **(K)** The protein level of c-Myc was assessed by Western blot in DTX-resistant PCa cells and their sensitive cells. **(L)** The relative protein level of c-Myc was evaluated in PC3 cells after transfected with PCAT1 wild-type or mutant plasmids. **(M)** Western blot was used to detect the c-Myc protein level in PCAT1-overexpressed PC3 cells under protein synthesis inhibitor CHX treatment. **(N)** The effect of proteasome inhibitor Borz on the change in the c-Myc protein level mediated by PCAT1 knockdown as detected by Western blot The data are presented as the means ± S.D. of at least three independent experiments. *P < 0.05.

Previous studies have pointed out that lncRNAs regulate gene expression functions by interacting with certain cellular proteins (22). Subsequently, we investigated whether *PCAT1* may also interact with specific proteins to regulate ferroptosis in DTX-resistant PCa cells. RNA pulldown and silver staining assays revealed that *PCAT1* binds several specific proteins with molecular weights of about 55 kDa in PC3/DR cells (**Figure 5A**). Among these proteins, we found that c-Myc was pulled down by the biotin-labeled probe *PCAT1* (**Figure 5B**). Moreover, an RNA Immunoprecipitation (RIP) assay confirmed the interaction between *PCAT1* and c-Myc in PC3/DR cells (**Figure 5C**). Next, we used two bioinformatic websites (i.e., CISBP-RNA and catRAPID) to investigate which domain of c-Myc contributes to the interaction with *PCAT1*. The results of catRAPID fragments revealed that the 1093-1367 nucleotide (nt) positions of the *PCAT1* sequence may bind to the 151-202 amino acid (aa) residues of the c-Myc protein with the top three propensities (**Figures 5D, E** **)**. To identify which c-Myc regions interact with *PCAT1 in vitro*, we constructed four different deletion fragments of the c-Myc protein (**Figure 5F**). RIP assays confirmed that *PCAT1* could bind to fragment 2 of c-Myc, which matched the predicted binding sites (**Figure 5G**). In addition, we also constructed a short biotin-labeled probe containing 1093–1367 nt of *PCAT1*, and the results confirmed that the above short probe bound to the c-Myc protein by RNA pulldown assay, indicating that 1093–1367 nt of *PCAT1* interacts with 151-202 aa of the c-Myc protein (**Figure 5H**).

Previous studies reported that *PCAT1* regulates c-Myc expression at the posttranscriptional level (23, 24). Consistent with the above results, we found that OE of *PCAT1* promoted the protein level of c-Myc in PC3 and 22RV1 cells, while *PCAT1* KD inhibited the c-Myc levels in PC3/DR and 22RV1/DR cells (**Figures 5I, J** **)**. As expected, no significant difference was found in the mRNA level of c-Myc upon *PCAT1* KD or OE (data not shown). In addition, DTX-resistant PCa cells had a higher expression of c-Myc protein than their sensitive cells (**Figure 5K**). To further identify the possible mechanism, we constructed *PCAT1* plasmids with deletion of predicted binding regions with c-Myc. As shown in **Figure 5L**, the protein level of c-Myc was increased in PC3 cells transfected with wild-type *PCAT1* but not the deletion form. To evaluate whether *PCAT1* was associated with the stabilization of the c-Myc protein, we treated PC3 cells with the protein synthesis inhibitor Cycloheximide (CHX) after *PCAT1* transfection. The results showed that the half-life of the c-Myc protein was significantly longer in *PCAT1* wild-type cells than in control cells (**Figure 5M**). Additionally, we treated *PCAT1*-KD cells with the proteasome inhibitor Bortezomib (Borz), and Western blot assays showed that Borz treatment reversed the inhibition of c-Myc protein upon *PCAT1* inhibition (**Figure 5N**). Collectively, these data demonstrated that *PCAT1* interacts and stabilizes the c-Myc protein.

### C-Myc Transcriptionally Promotes SLAC7A11 Expression to Inhibit Ferroptosis and DTX Resistance

**Figure 6:**
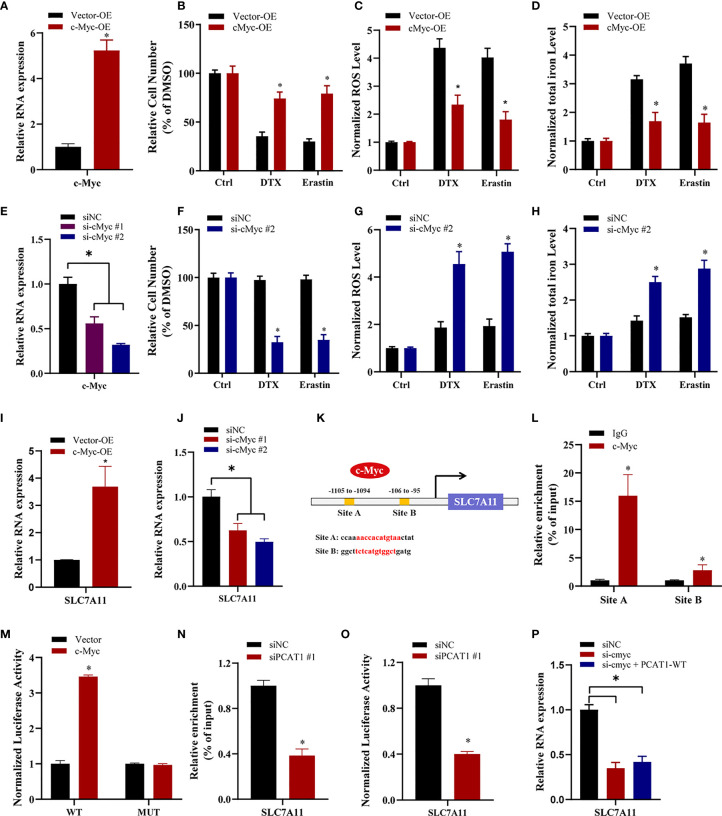
c-Myc transcriptionally promotes SLAC7A11 expression to inhibit ferroptosis and DTX resistance. **(A)** c-Myc overexpression PC3 cells were successfully established. **(B–D)** The cell viability, lipid ROS content and total iron concentration were assessed in PC3 cells after c-Myc overexpression upon DTX and erastin treatment. **(E)** c-Myc level was successfully reduced in PC3/DR cells by transfecting si-cMyc and control siRNA. **(F–H)** The cell viability, lipid ROS content and total iron concentration were assessed in PC3/DR cells after c-Myc knockdown upon DTX and erastin exposure. **(I, J)** The mRNA levels of SLC7A11 was evaluated by qRT-PCR upon c-Myc overexpression or knockdown. **(K)** Schematic diagram showing the location of c-Myc binding sites on the promoter regions of SLC7A11. **(L)** ChIP-qPCR was used to detect the binding efficiency of c-Myc on the SLC7A11 promoter. **(M)** The luciferase activities were measured in c-Myc overexpressed PC3 cells after co-transfected with SLC7A11 wild-type (WT) and mutant (MUT) plasmids. **(N, O)** The binding ability of c-Myc on the SLC7A11 promoter and luciferase activities of SLC7A11-WT plasmids were detected in PC3/DR cells after PCAT1 knockdown, respectively. **(P)** qRT-PCR assay was used to detect the expression of SLC7A11 in c-Myc-knockdown PC3/DR cells after co-transfected with PCAT1 plasmids. The data are presented as the means ± S.D. of at least three independent experiments. *P < 0.05.

To elucidate the specific mechanism of c-Myc-mediated chemo- and ferroptotic- resistance, we performed the following experiments upon c-Myc OE or KD. As shown in **Figures 6A, B**, ectopic expression of c-Myc in PC3 cells reversed erastin-induced and DTX-induced cell growth inhibition. Additionally, lipid ROS production and intracellular iron levels were greatly decreased upon DTX and erastin treatment in PC3 cells after c-Myc OE (**Figures 6C, D** **)**. In contrast, inhibition of c-Myc resensitized PC3/DR cells to erastin or DTX (**Figures 6E, F** **)**. The lipid ROS levels and iron content were increased in c-Myc-KD PC3/DR cells under DTX and erastin exposure (**Figures 6G, H** **)**. To explain whether c-Myc transcriptionally regulates SLC7A11 expression in PCa, we assessed the mRNA expression of SLC7A11 upon c-Myc OE or KD. qRT-PCR assays revealed that OE of c-Myc increased the SLC7A11 mRNA level, while KD of c-Myc increased the SLC7A11 mRNA level (**Figures 6I, J** **)**. In addition, we further searched the JASPAR database and found two c-Myc binding sites (namely, Site A and Site B) within the promoter region of SLC7A11 (**Figure 6K**). Chromatin immunoprecipitation (ChIP) assays showed that c-Myc was enriched mainly at Site A of the SLC7A11 promoter (**Figure 6L**). Moreover, we constructed wild-type and mutant SLC7A11 promoter reporter plasmids. The results showed that OE of c-Myc promoted the luciferase activities of wild-type SLC7A11 but not the mutant form, indicating that SLC7A11 was transcriptionally regulated by c-Myc (**Figure 6M**). We next investigated the mechanism by which *PCAT1* regulates the expression of SLC7A11. As illustrated in **Figure 6N**, the binding ability of c-Myc in the promoter of SLC7A11 was significantly inhibited in PC3/DR cells upon *PCAT1* KD. Next, the luciferase reporter assay revealed that KD of *PCAT1* impaired the transcriptional level of the SLC7A11 promoter in PC3/DR cells (**Figure 6O**). Rescue experiments revealed that inhibition of c-Myc greatly decreased the mRNA levels of SLC7A11 in *PCAT1*-transfected PC3 cells (**Figure 6P**). Taken together, these results confirmed that c-Myc transcriptionally activates SLC7A11 expression by interacting with *PCAT1*.

### PCAT1 Facilitates SLC7A11 Expression by Competing for MiR-25-3p

**Figure 7:**
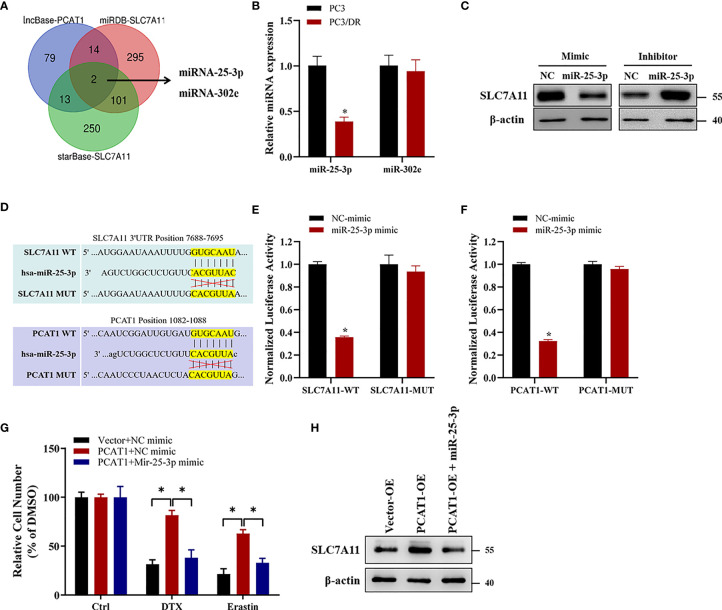
PCAT1 facilitates SLC7A11 expression by competing for miR-25-3p. **(A)** Venn diagram showing the possible target microRNAs of both PCAT1 and SLC7A11 by three online databases (i.e., lncBase, miRDB and StarBase). **(B)** qRT-PCR assay was used to detect the expression of miR-25-3p and miR-302e in PC3/DR and PC3 cells. **(C)** The protein levels of SLC7A11 in PC3/DR cells after transfected with miR-25-3p mimic and in PC3 cells after transfected with miR-25-3p inhibitor. **(D)** Schematic of SLC7A11 and PCAT1 wild-type (WT) and mutant (MUT) luciferase reporter vectors. **(E)** Relative luciferase activities were evaluated in PC3/DR cells co-transfected with SLC7A11 WT and MUT and miR-25-3p mimics and negative control. **(F)** Relative luciferase activities were analyzed in PC3/DR cells co-transfected with PCAT1 WT and MUT and miR-25-3p mimics and negative control. **(G)** The cell viability was assessed by CCK-8 assay in PCAT1-overexpressed PC3 cells after transfected with miR-25-3p mimic and negative control. **(H)** The protein level of SLC7A11 was detected by Western blot in PCAT1-overexpressed PC3 cells after transfected with miR-25-3p mimic and negative control. The data are presented as the means ± S.D. of at least three independent experiments. *P < 0.05.

Previous studies have indicated that *PCAT1* can function as a competing endogenous RNA (ceRNA) to regulate mRNA expression by sponging miRNAs (25). To identify the possible target miRNAs of both *PCAT1* and SLC7A11, we searched three online databases (i.e., lncBase, miRDB and StarBase). The results showed that miR-25-3p and miR-302e might putatively bind to *PCAT1* and SLC7A11 (**Figure 7A**). Subsequently, qRT-PCR assays showed that the expression of miR-25-3p was greatly downregulated in PC3/DR cells compared to PC3 cells, whereas no significant difference was found in the expression of miR-302e (**Figure 7B**). Western blot assays revealed that OE of miR-25-3p decreased the protein level of SLC7A11, whereas KD of miR-25-3p increased the SLC7A11 protein levels (**Figure 7C**). To further elucidate the interaction between *PCAT1*, SLC7A11 and miR-25-3p, we constructed *PCAT1* and SLC7A11 luciferase reporter plasmids containing the wild-type and mutated miR-25-3p-binding site sequences, respectively (**Figure 7D**). As shown in **Figure 7E**, a significant decrease in luciferase activities was observed following the transfection of miR-25-3p and wild-type *PCAT1* but not mutant *PCAT1*. Moreover, OE of miR-25-3p inhibited the luciferase activities after cotransfection of wild-type SLC7A11 plasmids but not the mutant form (**Figure 7F**). To further clarify whether *PCAT1* regulates SLC7A11 by sponging miR-25-3p, we performed rescue experiments following transfection with *PCAT1* and miR-25-3p in PC3 cells. The results showed that ectopic expression of miR-25-3p in *PCAT1*-overexpressing PC3 cells increased erastin-induced and DTX-induced cytotoxicity (**Figure 7G**). Additionally, the elevated protein level of SLC7A11 induced by *PCAT1* in PC3 cells was inhibited after transfection with the miR-25-3p mimic (**Figure 7H**). These findings together indicated that *PCAT1* regulates SLC7A11 expression by sponging miR-25-3p.

### TFAP2C Transcriptionally Activates PCAT1 Expression In DTX-Resistant Cells

**Figure 8:**
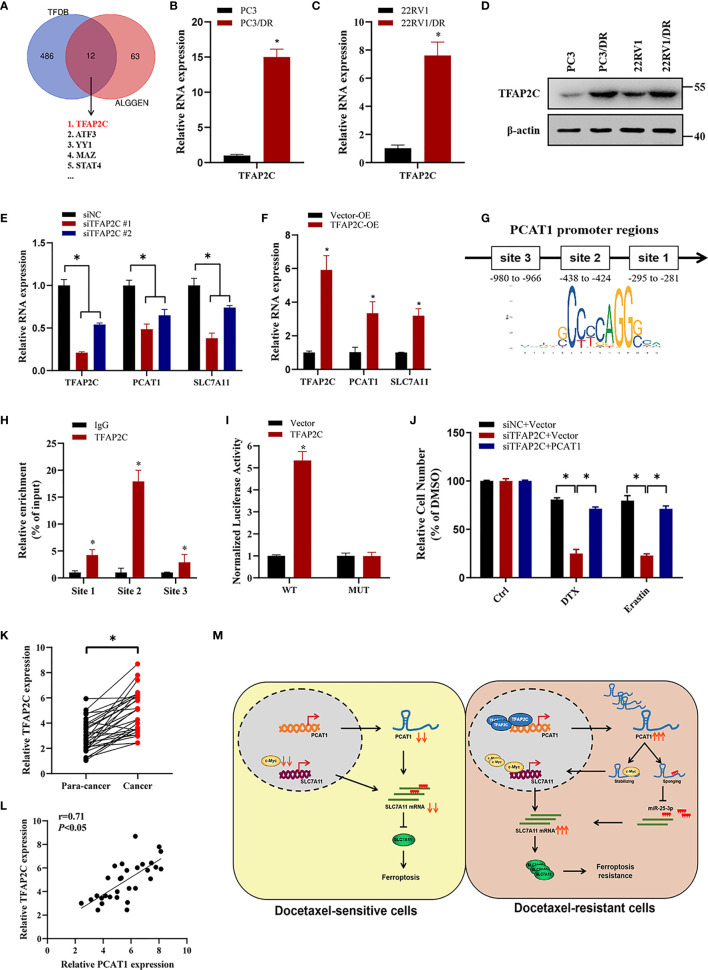
TFAP2C transcriptionally activates PCAT1 expression in DTX-resistant cells. **(A)** Venn diagram showing the possible transcription factors within the promoter regions of PCAT1 by online bioinformatics analysis (HUMAN TFDB and ALGGEN). **(B-D)** The mRNA and protein levels of TFAP2C were detected by qRT-PCR and Western blot in DTX-resistant PCa cells and their sensitive cells. **(E)** The mRNA levels of TFAP2C, PCAT1 and SLC7A11 in PC3/DR cells were detected after transfected with siTFAP2C and control siRNA. **(F)** qRT-PCR assay was used to assess the expression of TFAP2C, PCAT1 and SLC7A11 in PC3 cells after TFAP2C overexpression. **(G)** Schematic diagram showing location of TFAP2C binding sites on the promoter regions of PCAT1. **(H)** ChIP-qPCR was used to detect the binding efficiency of TFAP2C on the PCAT1 promoter. **(I)** The luciferase activities were measured in TFAP2C overexpressed PC3 cells after co-transfected with PCAT1 wild-type and mutant reporter plasmids. **(J)** Cell viability was assessed by CCK-8 assay in TFAP2C-knockdown PC3 cells after transfected with PCAT1 and vector plasmids. **(K)** The relative expression of TFAP2C was measured by qRT-PCR in 30 pairs of clinical PCa samples. **(L)** The positive relation between TFAP2C and PCAT1 in clinical samples (n=30) by qRT-PCR. **(M)** The schematic diagram showing that TFAP2C-induced PCAT1 inhibits ferroptosis in DTX-resistant PCa cells through c-Myc/miR-25-3p/SLC7A11 signaling. The data are presented as the mean ± SD, *P < 0.05.

To investigate the transcription factors that were responsible for the upregulation of *PCAT1*, we searched the possible transcription factors within the promoter regions of *PCAT1* by online bioinformatics analysis (HUMAN TFDB and ALGGEN) (**Figure 8A**). Among these databases, we found that TFAP2C was reported previously to be a ferroptotic factor (26). Then, we measured the expression level of TFAP2C in two DTX-resistant PCa cell lines and their parental cells. As shown in **Figures 8B–D**, the mRNA and protein levels of TFAP2C were greatly increased in PC3/DR and 22RV1/DR cells compared to PC3 and 22RV1 cells. KD of TFAP2C efficiently inhibited the levels of *PCAT1* and SLC7A11 in PC3/DR and 22RV1/DR cells, while TFAP2C OE activated *PCAT1* and SLC7A11 mRNA levels in PC3 and 22RV1 cells (**Figures 8E, F** **)**. Moreover, JASPAR predicted that there are three potential binding sites in the promoter regions of *PCAT1* (**Figure 8G**). Using a ChIP assay, we found the affinity of the TFAP2C protein to binding site 2 of the *PCAT1* promoter compared to other binding sites (site 1 and site 3) (**Figure 8H**). To further validate the direct transcription of TFAP2C to *PCAT1*, we constructed wild-type and mutant luciferase reporter plasmids targeting putative binding site 2. The results showed that transfection of the TFAP2C wild-type plasmid increased the relative luciferase activity of the *PCAT1* promoter, but transfection of the mutant TFAP2C binding sites did not (**Figure 8I**). In addition, KD of TFAP2C enhanced the cytotoxicity of erastin or DTX in PC3/DR and 22RV1/DR cells, whereas ectopic expression of *PCAT1* in TFAP2C-deleted cells rescued the above phenotype (**Figure 8J**). Our clinical data also indicated that TFAP2C was dramatically elevated in PCa samples compared with adjacent normal tissues (**Figure 8K**). The expression of TFAP2C was positively correlated with *PCAT1* levels in PCa samples (**Figure 8L**). These findings revealed that TFAP2C transcriptionally activates *PCAT1* expression, thereby contributing to DTX resistance in PCa cells (**Figure 8M**).

## Discussion

Ferroptosis is an iron-dependent programmed cell death induced by excess lipid peroxidation and accumulated lipid ROS in cells. Previous studies reported that high mesenchymal therapy-resistant persister cancer cells are vulnerable to GPX4 inhibition and ferroptosis induction (27). However, cisplatin was found to be an inducer of both ferroptosis and apoptosis, and cisplatin-resistant head and neck cancer was insensitive to ferroptosis because of Nrf2-antioxidant response element pathway activation (28, 29). In the context of PCa, ferroptosis induction is a new therapeutic strategy for castration-resistant, neuroendocrine and double-negative PCa as a monotherapy and in combination with second-generation antiandrogens (9, 10). At the beginning of this study, we deliberated to choose three hormone-resistant PCa cell lines (PC3, DU145 and 22RV1) to generate DTX-resistant cell lines by stepwise increased concentrations of DTX over a period of 6-9 months. Among them, PC3 and DU145 cells are androgen receptor -negative cells, while 22RV1 expresses AR-V7 which renders androgen independence. Subsequently, we used above three PCa cell lines (PC3, 22RV1 and DU145) to detect susceptibility to taxane and erastin. Consistent with previously described results, exposure to DTX could induce ferroptotic cell death in above three PCa cells (9). Interestingly, we also found that the cytotoxicity of erastin was greatly restrained in both PC3/DR and 22RV1/DR cells when compared to those of PC3 and 22RV1 cells. However, there was no significant change of cell growth between DU145 and DU145/DR cells after erastin treatment, indicating other ferroptosis’ pathway may contribute to the DTX resistance of DU145 cells. Thus, we chose PC3 and 22RV1 cells to further elucidate the potential role of ferroptosis in DTX resistance. Previous studies have demonstrated that erastin exposure inhibits the import of cystine though SLC7A11, resulting in suppression of GSH synthesis. Besides, erastin is also implicated in iron absorption and accumulation, which results in the synthesis of ROS and lipid peroxidase (30). Subsequently, we evaluated the phenotype of ferroptosis in DTX-resistant PCa cells upon erastin treatment, and the results showed that DTX-resistant PCa cells acquired ferroptosis tolerance. According to the above phenomena, we thought the possible mechanism was that long-term, low-dose DTX exposure increased the adaptive ability of oxidative stress and iron overload, which ultimately caused the chemoresistance and ferroptosis resistance of PCa cells (18). However, whether DTX-resistant PCa cells are also insensitive towards other ferroptosis inducers (i.e. RSL3, INF-γ, statins) remains unknown and should be further investigated.

In accordance with our findings, Zhou and colleagues found that OE of the drug efflux transporter ABCB1 in ovarian cancer cells led to DTX and erastin resistance (31). They found that erastin slightly inhibits the drug efflux activity of ABCB1 by evaluating intracellular rhodamine 123 accumulation, whereas the protein expression of ABCB1 was unchanged upon erastin treatment in ABCB1-overexpressing cells. Under these circumstances, we believe that there might be another pathway to regulate ferroptosis in DTX-resistant PCa cells. In the present study, we performed RNA sequencing of DTX-resistant and -sensitive PCa cells and further verified them by using qRT-PCR assay. On the basis of predefined inclusion and exclusion criteria, we selected the top 20 upregulated lncRNAs further analysis. Among these lncRNAs, PCAT1 is a ~1 900 nt lncRNA that is polyadenylated, localized to chromosome 8q24 and expressed in neoplastic, which was found significantly overexpressed in PCa tissues (25). Our previous study demonstrated that *PCAT1* activates AKT and NF-κB signaling in castration-resistant PCa cells by regulating the PHLPP/FKBP51/IKKα complex (21). However, whether *PCAT1* is involved in regulating ferroptosis in chemoresistant PCa cells has not yet been reported. Here, we found that *PCAT1* was robustly upregulated in DTX-resistant PCa cells and clinical samples. In addition, patients with higher serum *PCAT1* levels had a poor response to DTX chemotherapy. Besides, we further revealed that OE of *PCAT1* decreased erastin-induced and DTX-induced cytotoxicity in PCa cells. In contrast, the cell growth inhibition of erastin or DTX was restrained in PC3/DR and 22RV1/DR cells upon erastin or DTX treatment after *PCAT1* KD, with elevated lipid ROS content and iron overload. In xenograft experiments, we found that KD of *PCAT1* greatly resensitized DTX-resistant PCa cells to erastin or DTX. These combined data indicated that *PCAT1* could inhibit ferroptosis in DTX-resistant PCa cells *in vitro* and *in vivo*.

Next, we also explored the possible molecular mechanism of *PCAT1*-mediated ferroptosis in DTX-resistant PCa cells. RNA-protein pulldown assays revealed that *PCAT1* could interact with the c-Myc protein. Further experiments identified that 1093–1367 nt of *PCAT1* interacts with 151-202aa of the c-Myc protein. Mirrored with a previous study, inhibition of *PCAT1* decreased the levels of c-Myc protein but not the mRNA level (24). Then, we speculated that *PCAT1* could interact and stabilize with the c-Myc protein. Western blot analysis showed that OE of *PCAT1* inhibited the degradation of c-Myc protein, and proteasome inhibitor inhibited the inhibition of c-Myc protein in *PCAT1*-KD cells. Moreover, we also found that elevated expression of c-Myc transcriptionally increased SLC7A11 expression to regulate the ferroptosis process. Therefore, we demonstrated that *PCAT1* interacts with c-Myc and prevents its degradation, thereby promoting SLC7A11 expression. However, the specific degradation pathways of c-Myc in PCAT1-induced DTX-resistant PCa cells are still unexplored and should be further elucidated. In addition the binding proteins, *PCAT1* has also been found to be a competing endogenous RNA. Subsequently, using bioinformatic approaches, we predicted candidate miRNAs with sequences complementary to *PCAT1* and SLC7A11. By using loss- and gain-of function experiments and luciferase reporter assays, we identified that miR-25-3p directly targeted SLC7A11 and *PCAT1*. To date, some studies have revealed the biological function of miRNAs in chemoresistance and ferroptosis, such as miR-4443, miR-324-3p and miR-522 (32–34). In this study, ectopic expression of miR-25-3p inhibited the cytotoxicity of erastin or DTX in *PCAT1*-transfected PC3 cells. In addition, we also found that elevated SLC7A11 expression was inhibited in PCAT-OE PC3 cells after transfection with the miR-25-3p mimic. Together, these data demonstrated that *PCAT1* inhibits SLC7A11-mediated ferroptosis by sponging miR-25-3p.

To determine the reason for elevated *PCAT1* in DTX-resistant PCa, we predicted the transcription factors in the promoter of *PCAT1* regions. Then, we overlapped the above predicted possible transcription factors with ferroptosis-related factors (26). Among these factors, TFAP2C has been identified as the possible transcriptional gene of *PCAT1* in DTX-resistant PCa cells. Previous studies demonstrated that TFAP2C is vital for the regulation of gene expression during early development and the carcinogenesis process (35). In breast cancer, TFAP2C has multiple functions in regulating the expression of GPX4 in response to selenium supplementation (36). Besides, Chen and colleagues found that TFAP2C could activate the expression of MALAT1 and thus modulate the chemoresistance of DTX-resistant lung adenocarcinoma cells (37). In the present study, ChIP-qPCR and luciferase assays revealed that TFAP2C can directly bind to the promoter regions of *PCAT1* and activate the expression of *PCAT1* at the transcriptional level. Moreover, ectopic expression of TFAP2C disrupts the cytotoxicity induced by erastin or DTX, whereas inhibition of TFAP2C increases susceptibility to erastin- and DTX-induced ferroptosis. Together, the above data indicated that TFAP2C activates the expression of *PCAT1* transcriptionally, thus mediating chemoresistance and ferroptosis resistance.

## Conclusions

In summary, our study demonstrates that DTX-resistant PCa cells develop tolerance toward ferroptosis and that lncRNA *PCAT1* promotes chemoresistance by blocking DTX-induced ferroptosis. Mechanistic studies indicated that *PCAT1* activates the expression of SLC7A11 by interacting with c-Myc and sponging with miR-25-3p. In addition, TFAP2C activates *PCAT1* expression to reduce ferroptosis susceptibility and enhance chemoresistance. Collectively, these findings provide new insights into the roles of lncRNAs in ferroptosis and DTX resistance in PCa and demonstrate the potential of *PCAT1* as an effective target for PCa chemoresistance.

## Data Availability Statement

The raw data supporting the conclusions of this article will be made available by the authors, without undue reservation.

## Ethics Statement

The studies involving human participants were reviewed and approved by the Ethics Committee of the Second Hospital of Tianjin Medical University. The patients/participants provided their written informed consent to participate in this study. The animal study was reviewed and approved by the Ethics Committee of the Second Hospital of Tianjin Medical University.

## Author Contributions

XJ and YZ designed the study and wrote the manuscript. XJ, YZ, SG, and MX performed the majority of the experiments and analyzed the data. BM, RL, and YX provided the clinical samples and data. All authors have read and approved the final manuscript.

## Funding

This work was funded by grants from the National Natural Science Foundation of China (81802568 and 82174174), the Tianjin Municipal Science and Technology Project (20JCQNJC00530 and 20JCQNJC00550), and the Key Clinical Research Project of the Second Hospital of Tianjin Medical University (2018ZDSYS12 and 2019LC04).

## Conflict of Interest

The authors declare that the research was conducted in the absence of any commercial or financial relationships that could be construed as a potential conflict of interest.

## Publisher’s Note

All claims expressed in this article are solely those of the authors and do not necessarily represent those of their affiliated organizations, or those of the publisher, the editors and the reviewers. Any product that may be evaluated in this article, or claim that may be made by its manufacturer, is not guaranteed or endorsed by the publisher.

## Supplementary Material

Supplementary Table S1 The sequences of primers, oligonucleotides and probes used in this study.

Supplementary Figure S1 Verification of DTX-resistant PCa cell lines by CCK-8 and colony formation assays. **(A, B)** CCK-8 assay was used to detect the cytotoxicty of DTX in DTX-resistant PCa cells (PC3/DR and 22RV1/DR) and their parental sensitive cells (PC3 and 22RV1). Black line specifies the IC50 concentration of the drug for sensitive and resistant cells. **(C, D)** Colony formation assay was used to evaluate the cell growth in DTX-resistant PCa cells and their sensitive cells treated continuously for 14 days with DTX. The data are presented as the mean ± S.D. of at least three independent experiments. **P* < 0.05.

Supplementary Figure S2 Identification of top 20 upregulated lncRNAs in DTX-resistant and sensitive PCa cells. **(A)** Top 20 upregulated lncRNAs were selected from the RNA-sequencing data according to following criteria: fold change > 3, <5 000bp in length, baseline levels of TMM > 1.

Supplementary Figure S3 DTX-resistant PCa cells develop tolerance toward ferroptosis. **(A)** The relative expression of PCAT1 was evaluated by qRT-PCR in shPCAT1 #1 group and shNC group. The data are presented as the mean ± S.D. of at least three independent experiments. *P < 0.05.
