# Supplementary figures and images for: TFAP2C-Mediated lncRNA PCAT1 Inhibits Ferroptosis in Docetaxel-Resistant Prostate Cancer Through c-Myc/miR-25-3p/SLC7A11 Signaling
Source: Front Oncol. 2022 Mar 23;12:862015. doi: 10.3389/fonc.2022.862015 (PMC8985761; doi:10.3389/fonc.2022.862015)

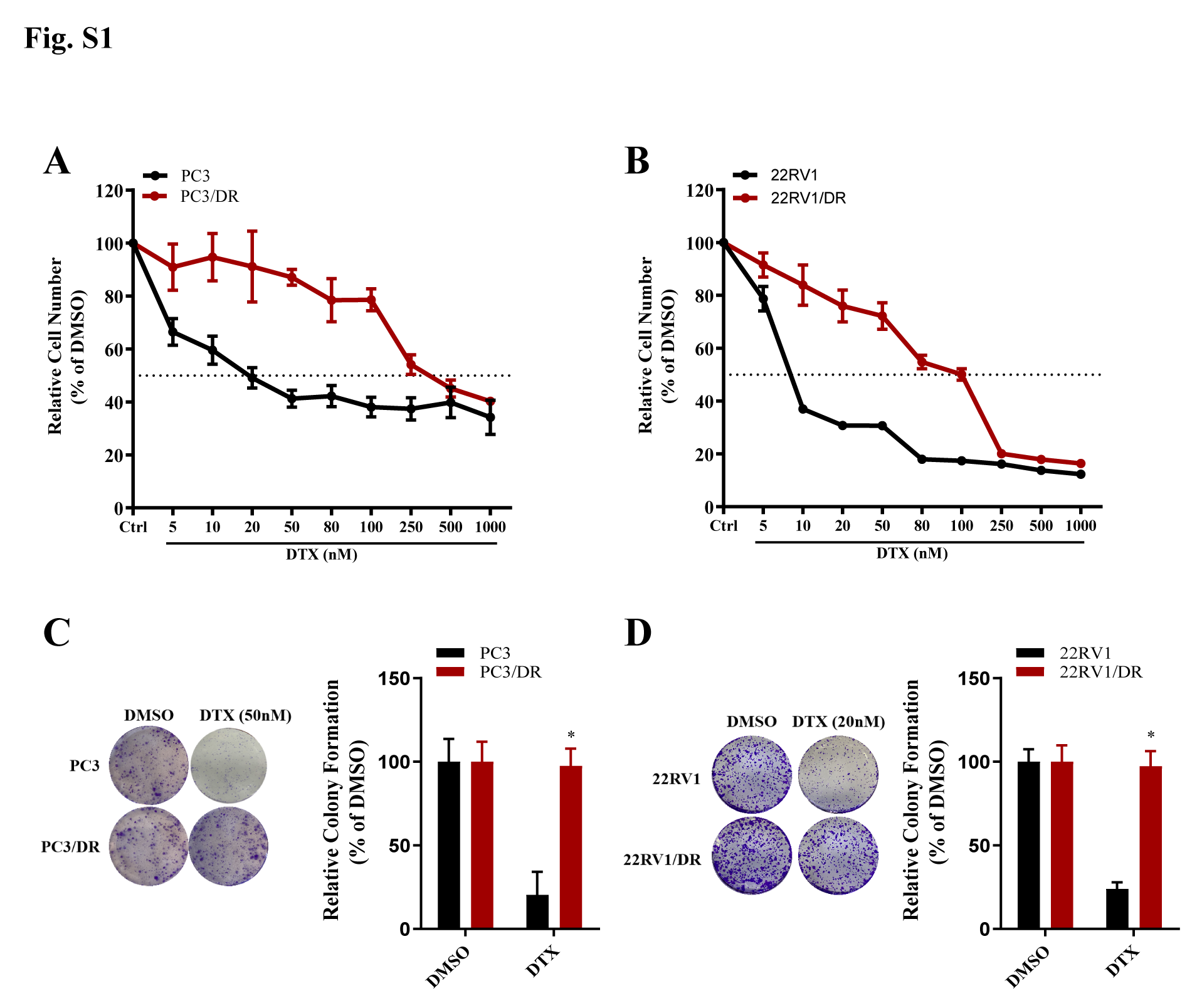

Supplement: Supplementary Figure S1 — Verification of DTX-resistant PCa cell lines by CCK-8 and colony formation assays. (A, B) CCK-8 assay was used to detect the cytotoxicty of DTX in DTX-resistant PCa cells (PC3/DR and 22RV1/DR) and their parental sensitive cells (PC3 and 22RV1). Black line specifies the IC50 concentration of the drug for sensitive and resistant cells. (C, D) Colony formation assay was used to evaluate the cell growth in DTX-resistant PCa cells and their sensitive cells treated continuously for 14 days with DTX. The data are presented as the mean ± S.D. of at least three independent experiments. *P < 0.05. [file Image_1.tif]

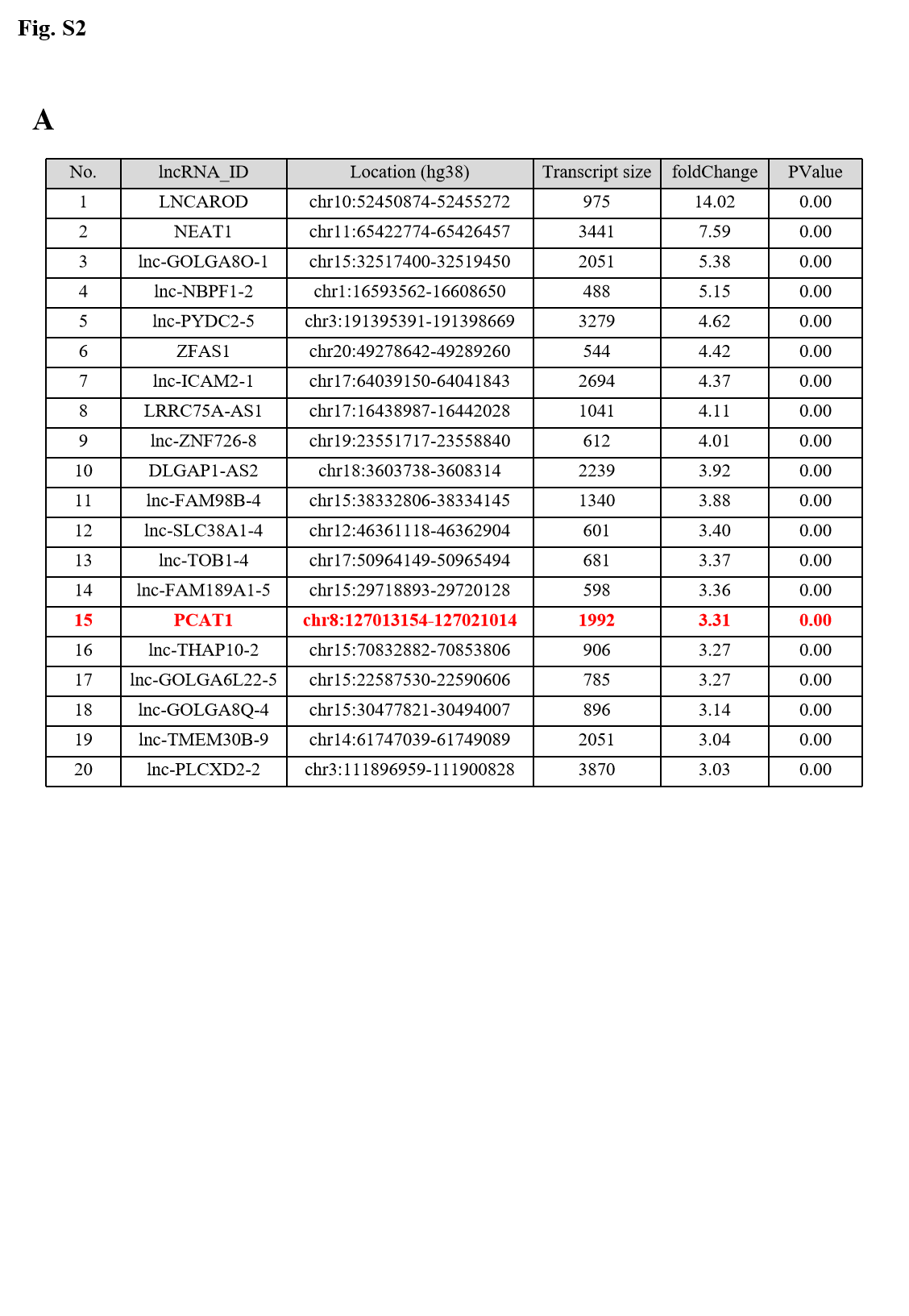

Supplement: Supplementary Figure S2 — Identification of top 20 upregulated lncRNAs in DTX-resistant and sensitive PCa cells. (A) Top 20 upregulated lncRNAs were selected from the RNA-sequencing data according to following criteria: fold change > 3, <5 000bp in length, baseline levels of TMM > 1. [file Image_2.tif]

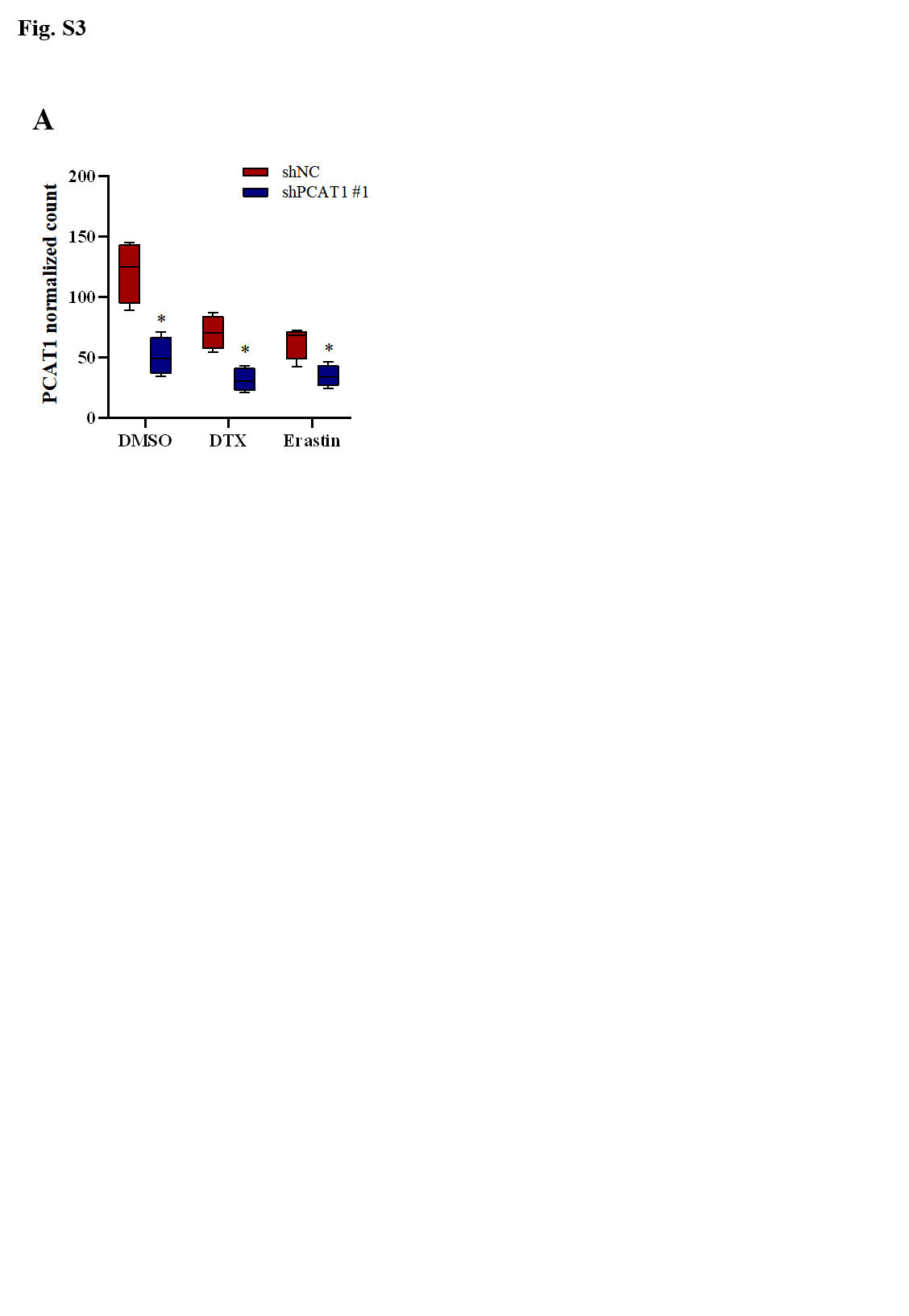

Supplement: Supplementary Figure S3 — DTX-resistant PCa cells develop tolerance toward ferroptosis. (A) The relative expression of PCAT1 was evaluated by qRT-PCR in shPCAT1 #1 group and shNC group. The data are presented as the mean ± S.D. of at least three independent experiments. *P < 0.05. [file Image_3.tif]
